# Polysaccharide-Based Bioink Formulation for 3D Bioprinting of an In Vitro Model of the Human Dermis

**DOI:** 10.3390/nano10040733

**Published:** 2020-04-11

**Authors:** Tanja Zidarič, Marko Milojević, Lidija Gradišnik, Karin Stana Kleinschek, Uroš Maver, Tina Maver

**Affiliations:** 1 Institute of Biomedical Sciences, Faculty of Medicine, University of Maribor, Taborska ulica 8, 2000 Maribor, Slovenia; marko.milojevic1@um.si (M.M.); lidija.gradisnik@um.si (L.G.); 2 Laboratory for Characterization and Processing of Polymers (LCPP), Faculty of Mechanical Engineering, University of Maribor, Smetanova ulica 17, 2000 Maribor, Slovenia; karin.stanakleinschek@tugraz.at; 3 Institute of Chemistry and Technology of Biobased Systems, Faculty of Technical Chemistry, Chemical and Process Engineering and Biotechnology, Graz University of Technology, Stremayrgasse 9, 8010 Graz, Austria; 4 Institute of Automation, Faculty of Electrical Engineering and Computer Science, University of Maribor, Koroška cesta 46, 2000 Maribor, Slovenia; 5 Department of Pharmacology, Faculty of Medicine, University of Maribor, Taborska ulica 8, 2000 Maribor, Slovenia

**Keywords:** alginate, carboxymethyl cellulose, nanofibrillated cellulose, human-derived skin fibroblasts, 3D cell-laden scaffolds, in vitro skin model

## Abstract

Limitations in wound management have prompted scientists to introduce bioprinting techniques for creating constructs that can address clinical problems. The bioprinting approach is renowned for its ability to spatially control the three-dimensional (3D) placement of cells, molecules, and biomaterials. These features provide new possibilities to enhance homology to native skin and improve functional outcomes. However, for the clinical value, the development of hydrogel bioink with refined printability and bioactive properties is needed. In this study, we combined the outstanding viscoelastic behavior of nanofibrillated cellulose (NFC) with the fast cross-linking ability of alginate (ALG), carboxymethyl cellulose (CMC), and encapsulated human-derived skin fibroblasts (hSF) to create a bioink for the 3D bioprinting of a dermis layer. The shear thinning behavior of hSF-laden bioink enables construction of 3D scaffolds with high cell density and homogeneous cell distribution. The obtained results demonstrated that hSF-laden bioink supports cellular activity of hSF (up to 29 days) while offering proper printability in a biologically relevant 3D environment, making it a promising tool for skin tissue engineering and drug testing applications.

## 1. Introduction

Skin, with its unique morphology, is critical for survival of the organism through acting as a functional barrier to a versatile range of environmental conditions, including stresses related to temperature changes, mechanical damage, and microorganisms [1,2,3]. Among its various vital functions, the human skin possesses highly effective self-regenerative properties, which is related to the presence of epidermal stem cells in different skin layers [1,4]. After damage, the skin activates its natural ability to promote spontaneous repair by following a four-stage process: hemostasis, inflammation, proliferation, and remodeling [4,5]. 

Loss of the epidermal barrier has serious adverse physiological effects. Open wounds present a high risk for fluid loss, high inflammation, bacterial colonization, and infection that can lead to sepsis. The reconstructed (e.g., scarred) tissue has a reduced functionality with loss of original properties of the uninjured skin [6]. Although a tremendous progress has been made over the past decades in the area of wound healing, autologous split-thickness skin grafts remain the gold standard in clinic for large wounds. However, there are several limitations of this approach. These include a shortage of donor skin tissue, as well as the potential additional health risks associated with the procedure (for example additional scarring, surgical site infections, hematoma, and bleeding) [7,8,9]. 

Lately, engineered skin substitutes present promising candidate products to alleviate these problems, and moreover, to induce a paradigm shift from using passive wound dressings to using bioactive cell-impregnated skin constructs [10]. Three-dimensional (3D) skin models have been under the spotlight for versatile applications in biomedicine, pharmaceutical, and cosmetic industries [3,10]. Beside skin grafts for wound healing, these have been proven as valuable in vitro models. Among others, they have been used to estimate skin permeability, to test potential adverse inflammatory responses that might occur during transdermal and topical drug invention, as well as served as important models to optimized drug formulations [11]. In contrast to animal skin, engineered skin exhibits several advantages, including better mimicking the human skin physiology, as well as mitigating ethical concerns and conforming to emerging regulations related to animal testing [11,12]. Furthermore, they serve as much better models (compared to animal models for example) to obtain fundamental insights into the etiology of skin diseases and related pathophysiological mechanisms associated with skin disease progression and treatment [13,14]. 

Although conventional tissue engineering methods have substantially contributed to clinical and dermatological studies, as well as facilitated the design and development of the first generation of skin grafts and models, at present, none of the bioprinted skin constructs can fully replicate native skin in terms of its morphological, biochemical, and physiological properties. However, simple skin constructs have already gained clinical value in burn and large wound treatment (e.g., Alloderm^®^, Biobrane^®^, Integra^®^) [3,7,10,11].

The inner micro- and macroscale features of the engineered skin tissue, which are prerequisite to accomplish satisfying aesthetic and functional results, have inspired scientists to employ additive manufacturing techniques that have already proven to be suitable for (basic) organ bioprinting and other tissue engineering strategies [3,10,11,15]. Bioprinting presents an advanced fabrication platform that enables preparation of 3D structured materials that allow a precise patterning of living cells and bioactive molecules (e.g., growth factors and cytokines) in predefined spatial locations [3,10,16]. By using computer-aided design (CAD), bioprinting has the potential to directly create graded macroscale architectures to better mimic the native extracellular matrix (ECM), therefore augmenting the attachment and proliferation of multiple types of cell concurrently. The 3D bioprinter has commonly the capability to dispense materials in X, Y, and Z directions, which facilitates the engineering of complex 3D structures in a bottom-up process. In addition, by utilizing multiple material platforms with various dispensing mechanisms, it enables incorporation of microfeatures to provide mechanical and biochemical cues at the microscale level that can further guide and enhance cellular alignment and differentiation. These characteristics assist the formation of intricate microenvironments and an architectural organization that can better satisfy various requirements of a natural niche for skin cells [10,16,17,18]. 

A critical step for (direct) bioprinting of a scaffold with incorporated cells lies in the selection and design of an ideal bioink. The latter must fulfill key requirements, including biocompatibility, desired viscoelastic behavior, a high mechanical integrity, and appropriate degradability. These properties are needed to achieve successful fabrication of complex biomimetic tissue substitutes. In addition, a bioink formulation also determines the selection of an appropriate bioprinting technique [18,19]. Typical bioinks incorporate a biocompatible hydrogel (bio)polymer solution and a suspension of desired cells. Hydrogels are appealing for cell encapsulation since they are mostly formed from polymers (e.g., polysaccharides, like alginate (ALG) and carboxymethyl cellulose (CMC)) that possess a favorable biocompatibility towards many cells. They provide structural support through direct contact with the cells, mimicking the natural ECM environment, as well as govern the chemical and physical properties of bioinks [16,20]. 

Current skin cell-printing approaches mostly rely on sequential printing of dermal fibroblasts and epidermal keratinocytes encapsulated in a homogeneous bioink, usually made from ALG, collagen, and/or fibrin [3]. Over the past decade, type I collagen has prevailed over other natural polymers as the most used base ingredient in skin tissue engineering [3,21,22]. Nevertheless, many studies have shown that for wound healing purposes, polysaccharides ALG and CMC also have a similarly positive effect, which is related to their similarity in many aspects to the natural skin ECM. These similarities include a good mechanical strength and stiffness, biocompatibility, and providing a highly hydrated environment, favorable for the skin [23,24,25,26,27]. Still, the application of hydrogel-based bioinks in fabrication of clinically relevant 3D skin constructs can be challenging, which is mostly related to their suboptimal printability for this purpose. Diverse bioink synthesis techniques that employ many materials and combination there-of enable amelioration of the bioink printability and as such can contribute to development of novel improved formulation for 3D bioprinting [28,29]. 

To compensate the inability to maintain a uniform 3D structure, hydrogels are often printed in combination with other materials. Recently, it has been demonstrated that shortcomings associated with using hydrogels, especially alginate-derived, can be overcome by adding cellulose nanofibrils [24,28,30]. Depending on the cellulose source and its processing conditions, these nanofibrills can be divided into three main categories: microfibrillated cellulose, nanocrystalline cellulose, and bacterial nanocellulose. All of these types of cellulose nanofibrils share the general properties of cellulose, such as hydrophilicity and a broad chemical modification capability, which are “upgraded” with properties that are specific for nanoscale materials (e.g., a high surface area). The positive effect of using nanofibrillated cellulose (NFC) in soft tissue engineering applications can be assigned to its (aforementioned) high potential hydration and its morphological similarity with ECM components (e.g., collagen) [18,28,30]. 

Utilization of nanocellulose materials (especially bacterial-derived) with its proven positive influence on adhesion, spreading and growth of both human-derived skin keratinocytes and fibroblasts, in skin regeneration and other areas of soft tissue engineering (e.g., carriers for cell delivery into skin defects) has been demonstrated by many studies [31].

Before clinical use of new therapeutics, the assessment of permeation of molecules is a crucial step in the prediction of in vivo performance and safety of dermal and transdermal drug delivery system. It is also an important quality-control measure to ensure batch-to-batch uniformity in pharmaceutical production. Prior to their commercialization, cosmetic products should be evaluated in terms of their efficiency, and potential allergic or even toxic effects. Incorporation of nanomaterials into numerous consumer products (e.g., textiles, medical devices, etc.) that could be in direct contact with human skin, raises additional questions of potential toxicity from dermal exposure. Thus, bioengineered skin models are also important investigation to determine the extent of penetration and of nanomaterials, but also several environmental pollutants and irritants [32,33,34,35]. Unique characteristics of human skin, such as pigmentation, dermal evolution, presence of adipose tissue, and skin appendage distribution, render skin behavior comparison between other mammalian species inaccurate. Since the structure and organization vary between mammalians, the penetration and absorption into the human skin cannot be completely predicted by studying them on animal models [35].

Based on everything mentioned, we developed a novel bioink that combines the shear thinning properties of NFC with the fast cross-linking ability of ALG and CMC. It was assumed that these characteristics make it possible to print complex skin constructs with a 3D architecture, resembling the skin native dermis, without the need of any additional supportive structures. In vitro assessments revealed a remarkable printability at a high-water content, cell viability, as well as favorable adhesion of keratinocytes, which even formed an epithelial layer similar to the native epidermis. Fabricated with a layer-by-layer deposition, 3D human-derived skin fibroblasts (hSF)-laden scaffolds displayed an increased shape fidelity, and promoted nutrient supply from the growth medium to encapsulated cells, favoring continuous growth of hSF. We examined closely the viability of the hSF cells encapsulated in the ALG/CMC/NFC-bioink after printing and cross-linking of the cell-laden scaffolds. In particular, the biological functionality of the as-formed bioink-based dermis model was shown in a 29-day in vitro 3D culture. Through this, it was suggested that the proposed preparation procedure leads to a highly promising dermal model, which can be further applied in skin regeneration.

## 2. Materials and Methods 

### 2.1. Materials

Sodium salt of alginic acid (ALG, Mw: 80 kDa), carboxymethyl cellulose (CMC, Mw: 700 kDa) and calcium chloride for bioink and subsequen scaffold production, as well as phosphate-buffered saline (PBS), l-glutamine, penicillin G sodium salt, streptomycin sulfate salt and the Live/Dead cell double staining kit, were all purchased from Sigma-Aldrich, Germany. A suspension of cellulose nanofibrils (NFC, 3% (*w*/*v*)) was acquired from The Process Development Center, University of Maine (Orono, ME, USA). Advanced Dulbecco’s Modified Eagle Medium (ADMEM), Advanced Dulbecco’s Modified Eagle Medium/Nutrient Mixture F-12 (ADMEM/F-12) and fetal bovine serum (FBS) were purchased from Thermo Fisher Scientific (Schwerte, Germany). Human derived skin fibroblasts (ATCC CCL-110, Detroit 551, LGC Standard) were obtained from LGC Standard, Bury, Lancashire, UK. Keratinocytes, an aneuploid immortal keratinocyte cell line (HACAT) from adult human skin, which were used to test the potential of the developed dermis model to grow also an epidermal layer on top, was kindly donated by Prof. Dr. Elsa Fabbretti (Centre for Biomedical Sciences and Engineering, University of Nova Gorica, Nova Gorica, Slovenia). All the materials were used as received without any further modification prior to sample preparation or testing. For preparation of calcium chloride solutions (and other water-based solutions), ultra-pure water (18.2 MΩ cm at 25 °C), prepared using an ELGA PURELAB water purification system (ELGA LabWater, Veolia Water Technologies, High Wycombe, UK), was used.

### 2.2. Culture of Human Skin Fibroblasts (hSF) for 3D Printing 

Human derived skin fibroblasts (hSF) were grown and maintained in ADMEM, supplemented with 5 wt.% FBS, 2 mmol/L l-glutamine, 100 U/mL penicillin and 1 mg/mL streptomycin (ADMEM + 5 wt.% FBS) 37 °C in a humidified 5 wt.% CO_2_ atmosphere in tissue culture flasks (Nunc^TM^, Thermo Fisher Scientific, Bremen, Germany) until confluent. The cell growth medium was routinely changed (every second day). After printing, 3D hSF-laden scaffolds were maintained ADMEM + 5 wt.% FBS with regular medium changes (every 2–3 days). Cells were imaged daily using an inverted optical microscope (Axiovert 40, Carl Zeiss Microscopy, Munich, Germany). For an estimation of viability of encapsulated hSF, 3D cell-laden scaffolds were removed from medium, rinsed with PBS (pH 7.4) and treated with Live/Dead solution (according to the manufacturer protocol). Finally, each 3D scaffold was observed and imaged under a fluorescence microscope (EVOS FL Cell Imaging System Thermo Fisher Scientific Inc., Germany).

### 2.3. Preparation of the Bioink (Formulation and Cells) for 3D Printing

**Figure 1:**
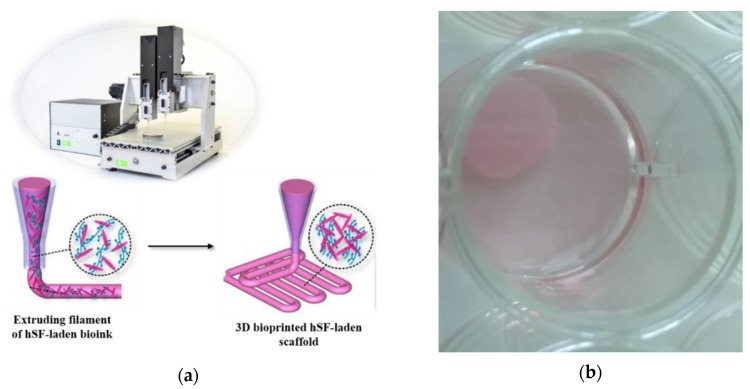
Fabrication of human-derived skin fibroblasts (hSF) cell-laden scaffolds: (**a**) Schematic presentation of 3D bioprinting of hSF-laden scaffold; (**b**) 3D bioprinted hSF-laden scaffold immersed in Advanced Dulbecco’s Modified Eagle Medium (ADMEM) + 5 wt.% fetal bovine serum (FBS). Additional photographs of the printed ink and bioink formulations, as well as the model used for the printing, are shown in Figure S1 of the Supplementary Materials.

Following the bioprinting process implemented by our group [36], 4-layer hSF cell-laden scaffolds with 3D architecture were achieved using a layer-by-layer fabrication approach (Figure 1). For this purpose, we used the VitaPrint 3D bioprinter (Institute IRNAS, Maribor, Slovenia), which enables the formation of pores of a chosen size, shape and pore density in z-direction, and extrusion nozzles (Nordon EFD, East Providence, RI, USA) with 0.25 mm diameter. Using a layer-by-layer approach subsequent layer were deposited in 0°/90° fashion on a glass surface. Before bioprinting was initiated, a cylinder-shaped scaffold with a 10 mm diameter and a height of 0.8 mm were modeled using the Autodesk software (Autodesk Inc., San Rafael, CA, USA). The pore size was pre-defined in a g-code generator software (Slic3r 1.2.9, open access program published under GNU Affero General Public License) [36] and was set to a constant 30% infill across the scaffold.

The formulation used in this study was previously partially optimized [24,36,37] and herein modified to serve the specific needs to prepare the bioink formulation (e.g., the water part was exchanged with the cell growth media, the concentration of the used calcium chloride solution for cross-linking had to be reduced, etc.). The preparation procedure was as follows. To prepare the bioink, we first mixed ALG and CMC in ADMEM + 5 wt.% FBS and 3 wt.% NFC to obtain the final formulation composed of 3 wt.% ALG + 3 wt.% CMC + 1.5 wt.% NFC. The as-prepared formulation was sterilized under UV light for 30 min. The composition had to be optimized in order to achieve a desired printability, leading structurally stable scaffolds with a preserved (as printed) geometry. Before their in situ incorporation into the bioink formulation, the hSFs cell pellets were re-suspended in ADMEM + 5 wt.% FBS. Then, we manually mixed 1ml of the suspension of hSF (10^6^ cells/mL) into the as prepared bioink (e.g., ALG/CMC/NFC) formulation.

After printing, which was performed under aseptic conditions in a laminar flow hood, all hSF-laden scaffolds were additionally cross-linked using a 2 wt.% CaCl_2_ solution. This “post-processing treatment” was done by pouring a 2 wt.% CaCl2 solution with a pipette onto the scaffold, completely covering it. The scaffold was left in the solution for 1 min, which was shown to be enough to allow for ionic gelation. After that, the scaffolds were carefully removed from the solution and moved into a 12-well plate. Finally, each hSF-laden scaffold in respective P12 plate wells were covered with ADMEM + 5 wt.% FBS. The whole P12 plates were rapidly transferred into an incubator operated at 37 °C and in a controlled 5 wt.% CO_2_ atmosphere. 

### 2.4. Rheological Measurements

The rheological properties of the bioink formulation was characterized using a Rheolab QC rheometer (Anton Paar, Graz, Austria) equipped with a cylinder measurement system CC27-SN25789 (Anton Paar, Austria) according to the ISO 3219 standard. During the measurements, the temperature was kept constant at 37 °C. For the determination of viscosity profiles, the shear rate (Γ) was continuously increased from 0.01 to 1000 s^−1^, comprising 31 measuring points per 10 s for a total duration of 310 s. The analysis method and curve fitting were performed according to the Carreau-Yasuda model [38]. 

### 2.5. Wettability (Hydrophilicity/Hydrophobicity)

The hydrophilicity of the bioink formulation was investigated using the water contact angle measurement method (CA), which was performed on an OCA15+ goniometer system (Dataphysics, Filderstadt, Germany). The sample masses (*m*), which change as a function of time (*t*) during the water adsorption phase, were monitored. The initial slope of the function *m*^2^ = *f* (*t*) is known as the capillary velocity, which can be used for determination of the contact angle between the solid (polymer sample) and water using a modified Washburn equation [26,39]. All measurements were executed on three independent samples at three different sample regions. For this purpose, a smear of the bioink formulation was prepared between two glass slides (in 3 replicates). An average value with the standard error was calculated. 

### 2.6. Water Uptake (Swelling Ratio)

The gravimetric method [40] was employed to understand the swelling kinetics of the bioink formulation. The dried 3D scaffolds were first weighed to determine initial weight (*W*_0_), and then immersed in ADMEM + 5 wt.% FBS (pH 7.4) at 37 °C. At predetermined time intervals, each scaffold was removed from ADMEM + 5 wt.% FBS, wiped dry with filter paper on the surface, and weighed. The swelling ratio at time *t* was calculated as:

(1) $$Swelling\;ratio=\frac{W_{t}-W_{0}}{W_{0}}\times 100\%,$$

where *W*_0_ and *W_t_* are the weights of the initial dry and swollen scaffolds at predetermined time *t*, respectively. All measurements were conducted in triplicates, and the average values were calculated and plotted together with the standard error.

### 2.7. In Vitro Degradation Test

For this testing the air-dried (dried for 24 h at room temperature at a RH of ~20% in a laminar) cylinder-shaped 3D bioink scaffolds (10 mm in diameter, 30 mm in height), without in situ incorporated cells, were first weighed (*W*_0_), and then immersed in ADMEM + 5 wt.% FBS (pH 7.4), followed by incubation at 37 °C in 5 wt.% CO_2_ atmosphere for varying times [40,41]. At predetermined time intervals, the scaffolds were removed from ADMEM and dried on (ambient) air. All the experiments were done under sterile conditions to prevent bacterial and fungal contamination. The remaining weight of the scaffold was calculated according to the equation:

(2) $$Weight\;remaining=\frac{W_{t}}{W_{0}}\times 100\%,$$

where *W*_0_ is the initial weight and *W_t_* is the remaining weight of the scaffold at predetermined time *t*. All measurements were carried out in triplicates, and the results are reported and plotted as average values with the standard error.

### 2.8. Live/Dead Assay of Human-Derived Skin Fibroblasts (hSF)

The 3D hSF-laden scaffolds (in triplicates) were fabricated as described above. The Live/Dead assay was performed as follows. At predetermined time points (2 h, 24 h, 48 h, 4 days, 6 days, 8 days, 12 days, 15 days, 25 days, and 29 days) the Live/Dead solution, containing calcein-AM and propidium iodide (PI) in PBS (pH 7.4), was used to replace the culture medium of each P12 well, hosting respective scaffolds. Next, the whole plate was incubated at 37 °C under a 5 wt.% CO_2_ atmosphere. Viability of the cells was evaluated by reviewing living (green) and dead (red) cells on micrographs that were randomly taken during scaffolds observation using a fluorescence microscope (EVOS FL Cell Imaging System Thermo Fisher Scientific Inc., Bremen, Germany). 

## 3. Results and Discussion

### 3.1. Printability of the Prepared Bioink Formulation

In general, printability refers to the relationship between the bioink formulation and its interaction with the surfaces it gets in touch with during printing. These include the container, from which the bioink is printed, and the substrate onto which the bioink is printed. This relationship is the basis for phenomena that govern the construction of an accurate, high-quality 3D pattern. As such, the printability is normally associated with the surface tension of the supporting structures. The latter crucially affects cell attachment, proliferation and differentiation. In fabrication of 3D scaffolds, a hydrogel matrix should maintain surface tension in the vertical direction, as well as possess a large contact angle with the substrate (for a hydrogel with a high hydrophilic character, a substrate should exhibit a “more” hydrophobic nature), i.e., deposited hydrogel filaments should not be “too flat” on the substrate [16]. Several factors that have a high impact on printability (e.g., resolution, shape fidelity, etc.) can be modulated through careful tuning of the formulation’s mechanical properties, among others, the viscoelastic behavior of material. Poor Z-layer definition compromises the overall printing “resolution”, which depends on the construction of multiple layers, and the ability for precise patterning of different materials, which is hampered by potential material spreading [29]. 

**Figure 2:**
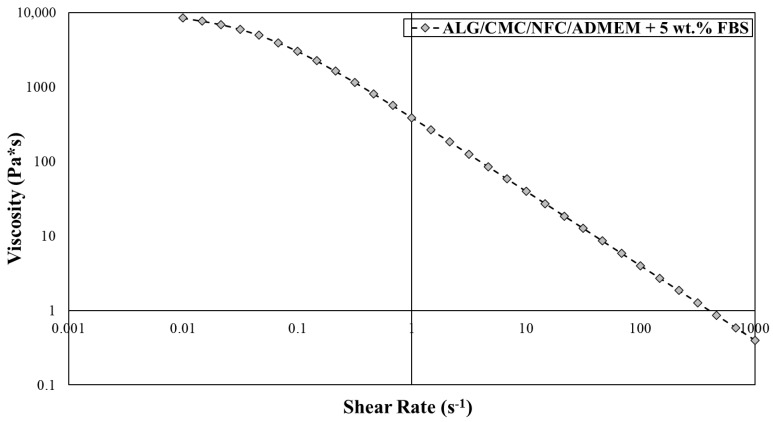
Viscosity measurements for alginate/carboxymethyl cellulose/nanofibrillated cellulose (ALG/CMC/NFC)-based bioink formulation using the Anton Paar Rheolab QC with cylinder measuring system CC27.

The extrusion-based 3D bioprinting approach relies on deposition of highly viscous (cell) solutions through nozzles, which can result in mechanical stresses (e.g., shear forces) that can damage encapsulated cells. Therefore, bioinks shear-thinning properties are necessary to compensate for these high shear stresses, results from the mentioned (desired) bioinks high viscosity. Herein, an already partially tested bioink formulation [24,36,37] was further optimized to serve the specific needs of skin model bioprinting. The crucial changes in the formulation had to include the exchange of the “water part” with the cell growth media (ADMEM + 5 wt.% FBS), as well as adjustments (decreasing) in the calcium chloride concentration, which was used for cross-linking. Prior to bioprinting of the 3D cell-laden scaffolds, the viscosity of the prepared bioink formulation had to be further optimized to ensure proper mixing with the cell suspension on one hand, and to achieve homogenous 3D distribution of encapsulated cells throughout the whole timeframe of the bioprinting procedure without compromising cell viability on the other [19]. After an optimized bioink formulation was prepared, 3D bioprinting (using a VitaPrint Bioprinter, Institute IRNAS, Maribor, Slovenia) was used to build defined, self-supportive scaffolds in a layer-by-layer fashion. To yield optimal layer-by-layer definition, which is prerequisite for bioprinting of self-supportive scaffolds, the flow properties of the prepared bioink formulation (without encapsulated hSF) were investigated (Figure 2). 

The obtained flow curve revealed a shear thinning behavior of the bioink formulation, for which the viscosity decreases with an increasing shear rate. In terms of the 3D bioprinting process, such behavior predisposes the bioinks smooth passage through the nozzle with a diminished risk for clogging it. In general, hydrogel precursors (e.g., ALG and CMC) normally display a time-dependent non-Newtonian behavior, for which the viscosity is dependent on shear rate, and the relation between the shear stress and the shear rate is different (the shear stress-shear curves show nonlinear, concave patterns) [19,42]. Considering the latter prior to preparation of the bioink formulation herein, a literature search revealed several recent studies, in which the authors demonstrated remarkable printability of nanocellulose/alginate derived hydrogels, which led to formation of biologically relevant 3D architectures, owing to their highly viscous and shear thinning nature [18,28]. Careful observation of Figure 2 further reveals that at a low shear rate (10 s^−1^), a higher viscosity is observed (viscosity value around 40 Pa s), whereas an increase in the shear rate to 500 s^−1^, results in sharply decreased viscosities around 1 Pa s. Our results are also in agreement with previous studies, like the one by Gillispie et al., which further claim that the extrudability can be further assessed using the Live/Dead assay (which will be discussed latter on in the article) [43].

To support cell proliferation after the bioprinting, the prepared bioink formulation must form a quasi-scaffold structure [16]. To meet with this requirement, a cross-linking post-printing treatment becomes essential. The prepared bioink formulation in this study includes polysaccharides ALG, CMC, and NFC. Both ALG and CMC are anionic polymers, which can be successfully (ionically) cross-linked with the addition of divalent Ca^2+^ ions that bind on functional groups, resulting in formation of ionic inter-chain bridges or calcium complexes. The concentration of calcium chloride has to be lowered compared to other studies [24,36] to prevent unnecessary influence on the cell growth. Post-printing processing was performed using a 2 wt.% CaCl_2_ solution, which further stabilizes the polysaccharide-based network present in the 3D bioprinted scaffold by interacting with binding sites (carboxyl groups presented along the ALG polymer chain) [27,44], and by forming calcium complexes (with CMC) to promote polymerization and the extent of lateral aggregation [44]. 

### 3.2. Wettability (Hydrophilicity/Hydrophobicity) of the Bioink Formulation

Surface wettability is one among the features contributing to the skin’s barrier function. The latter is regarded as an indicator of physiological similarity to an in vivo response [3,45]. Besides that, to aid wound healing process, a moist environment allows the cells to move unhindered outwards through the thin layer of the often present wound exudate [26]. To evaluate the wettability of the prepared bioink formulation, we evaluated the surface wettability through the water contact angle (CA(H_2_O)) measurements. The contact angle values directly reflect the surface wettability and are sensitive to chemical functionalities of the outermost layer [39,46]. The smaller the manifested (CA(H_2_O)), the better the wetting of the surface [47].

Since the proposed bioink formulation is composed from polysaccharides (e.g., ALG, CMC, and NFC, although there is some controversy in this regard as shown in [48], where NFC seemed to increase the contact angles of the base film composite samples), known for their hydrophilic nature, we expected that a bioink possesses high surface wettability. It is known that wettability, along with surface topography, determines cell/material interactions, whereas wettability itself is affected by material chemistry and topography. A general consensus exists that cells favor more hydrophilic surface to adhere and proliferate [49]. The obtained (CA(H_2_O)) values (lower <90°) for all samples confirm the hydrophilic character [39] of the ALG/CMC/NFC-based bioink formulation. According to the available literature, there is no exact water spreading extent on the human skin. Namely the spreading rate (which is directly related to its contact angle) of water on the skin varies from 57° to 92°, depending on the content of sebum present on the skin [47]. Our results (i.e., a mean (CA(H_2_O)) value of 70.9 ± 2.7°) present a promising result regarding the simulation of the physiological barrier function of the skin, although it has to be stated that the full recapitulation of this function would require a successful stratification and keratinization of the epidermal layer. This result is also in agreement with other literature sources, which report similar hydrophilic/hydrophobic properties and relate these with those of the skin-derived extracellular matrix reported by Kim et al. [3]. Furthermore, according to literature, the evaluation of surface wettability is one of the features that can be used to indicate the skin’s barrier function [3,45]. 

### 3.3. 3D Bioprinted Scaffolds Water Uptake Capacity (Swelling Ratio)

**Figure 3:**
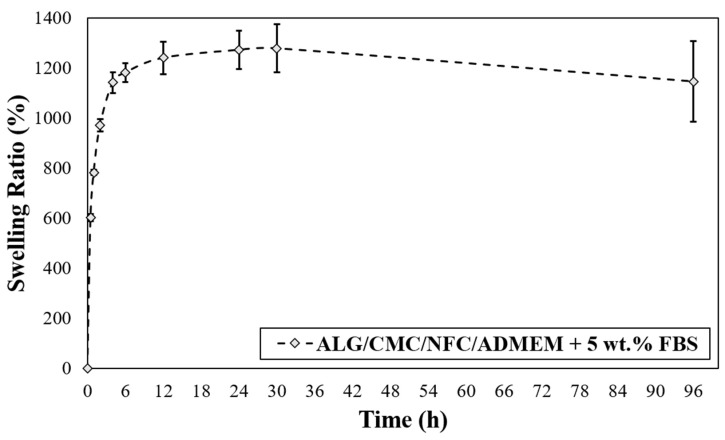
Scaffold swelling test for ALG/CMC/NFC-based bioink formulation.

The swelling properties of the hydrogels are among the most crucial parameters of tissue engineering. This is related to their influence on a wide range of parameters including various surface properties, as well as the extent of possible diffusion of incorporated and “environmental” signaling molecules and nutrients in a hydrogel [50]. Another critical parameter in bioprinting applications that is affected by the base formulations swelling properties is the printing resolution. The latter significantly affects the materials capability to accomplish an accurate (desired) spatial arrangement of materials and cells. In terms of swelling properties, if hydrogels swell too much, the intended spatial placement might be lost [51]. As can be seen from Figure 3, the prepared bioink-based 3D scaffolds exhibited an upward trend in the swelling rate, which persists over the course of the first 30 h of media exposure. The presence of many hydrophilic groups in ALG and CMC together with the possibly highly hydrated NFC lead to higher entry of water into the bioink formulation. In the case of the prepared ALG/CMC/NFC-based scaffolds a fast-swelling kinetics is evident, namely the scaffolds take up (swell) approximately 1200% of their initial weight. A “rather small” weight loss observed after 30 h suggests an increased permeability of the scaffold, which might facilitate degradation of scaffolds. 

### 3.4. In Vitro Degradation of the 3D Bioprinted Scaffolds

**Figure 4:**
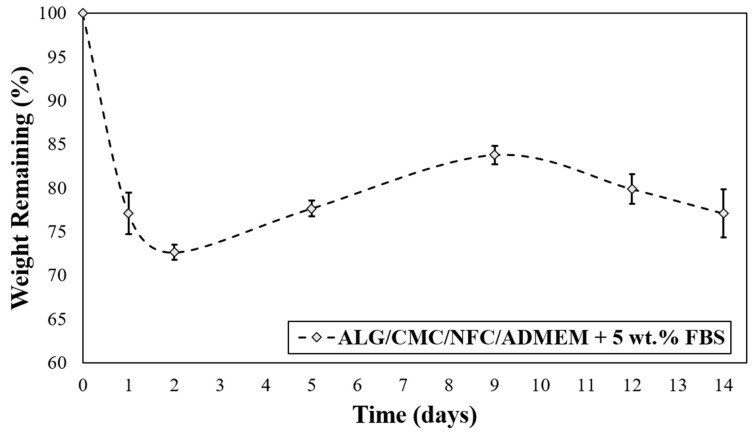
In vitro degradation rate for ALG/CMC/NFC-based bioink formulation.

The degradation rate of the 3D cell-laden scaffolds depends on the specific bioink material used and must be adapted according to the desired application. In general, an “optimized” 3D scaffold should provide mechanical support during tissue regeneration, and at the same time be degradable in vivo to allow tissue remodeling. Ideally, the degradation rate should match the rate of tissue regeneration, allowing for a gradual replacement of the biomaterial scaffold with newly formed ECM components of continuously growing cells during regeneration [19]. The investigation of the 3D printed ALG/CMC/NFC-based scaffolds degradation kinetics was carried out to evaluate whether the scaffolds can maintain structural integrity and have potential to support cell viability of encapsulated hSF for (at least) up to 14 days (Figure 4). 

Interestingly, after the initially observed weight loss (which is most likely the result of cation exchange between Na^+^ from growth median for Ca^2+^ from the scaffold that leads to dissolution of ALG), the scaffolds are regaining some weight during the following week (up to day 10), and finally start to lose mass at a slower rate. The mentioned weight gain between days 2–10 can probably be ascribed to an uptake of some ingredients from the cell growth media (ADMEM + 5 wt.% FBS (e.g., glucose, protein from FBS, glutamine, etc.)) that might have taken up the spaces in the scaffold structures that were vacated by the dissolved molecules of ALG. On the other hand, an initial weight loss of scaffolds can be further elucidated also with the corresponding swelling profile (Figure 3), described in the previous section. The swelling equilibrium that was reached after 30 h coincides with the lowest measured weight (Figure 4), implying a possible impairment of the ALG/CMC network [24] that loosened the “internal stress” of the bioink formulation, and consequently caused some deformability [52]. In turn, degradation of scaffolds could have enlarged internal voids, allowing further water uptake (and cell growth media, affecting even nutrient diffusion/distribution rates), which can in turn promote cell migration (more space for cells to move between these voids) and potentially also proliferation. Although the latter is not clearly evident from the SEM images (Figure S5 of the Supplementary Materials) apart from Figure S5b after 29 days, which shows larger distances between the respective internal scaffold walls (red arrow), the possible explanation is that the material surface remained intact, whereas the internal structure was re-organized, without affecting the overall stability of the materials. Further methods to prove the latter will need to be employed in future studies, with microCT (or even nanoCT) would be best suited for this purpose. Stiffer networks (in scaffolds), which are less prone to deform, may hinder cellular function. Since cells need sufficient space to proliferate, the reduction of the scaffolds lattice rigidity is vital for their spreading and proliferation [52,53]. 

To further evaluate the in vitro degradation of the prepared samples, were the obtained results compared to some recently published results of other research groups. For example, Barbeck et al. prepared scaffolds based on PLA and a calcium phosphate glass (even in a bi-layer form). Without the added glass particles, their scaffold degraded for approximately 9% over a period of 8 weeks [54]. Jin et al. combined theoretical modelling and actual scaffold preparation to show the difference between differently shaped types of scaffolds based on the same printed PLA/beta-TCP/HA base material. They found variable degradation extents among differently structured scaffolds, ranging from 5% to 25% of the initial mass [55]. Dong et al. prepared 3D-printed poly(ε-caprolactone) scaffolds with incorporated cell-laden chitosan hydrogels. While varying composition (only PCL, CHI or a hybrid formulation), they could tailor the degradation rate from 15% to 60% in the timeframe of 20 days [56]. Walker et al. 3D printed high resolution poly(propylene fumarate) scaffolds. They varied the input polymer molecular masses and used two architecture styles for the scaffold design. Through these, they were able to control the degradation of the prepared scaffolds in a range from 20% to almost 70%. Raddatz et al. prepared different three dimensional printed resorbable materials based on PLA, PLcoDLA, PCL, and PDO) and, among others, compared their in vitro degradation rate. They found a degradation rate in the range of 22–28% [57]. Although these examples show variable extents of the overall degradation rate (which is of course related to the chosen base material and potential additives, strengthening the overall scaffold structure), all have in common the stating of the importance of a stable enough internal scaffold structure, promoting the long-lasting growth of the incorporated (different) cells. The latter is also something we have found to be of utter importance for our studies.

In conclusion, despite the observed mass losses, even after 14 days the overall weight loss was not critical to jeopardize structural integrity of the scaffolds and hence confirms the prepared bioink-based scaffold promise for a long-term application.

### 3.5. Live/Dead Assay to Evaluate the Viability of the Cell-Laden 3D Bioprinted Scaffolds

The viability of the encapsulated hSF cells is under influence of many factors. These include the stiffness of the 3D bioprinted cell-laden scaffold, the used cross-linking approach, the bioinks hydrophilicity, as well as its viscosity. One of the vital prerequisites of the material formulation in a bioink, is to protect the encapsulated cells from damaging stresses experienced prior (physical mixing into the bioink formulation), during (shear forces during extrusion through the 3D printing nozzle), and after (cross-linking) 3D bioprinting. Therefore, a crucial step in investigation of the prepared bioinks suitability for bioprinting of viable cells, is to investigate the effects of the bioink and bioprinting itself on the cell viability [19]. 

**Figure 5:**
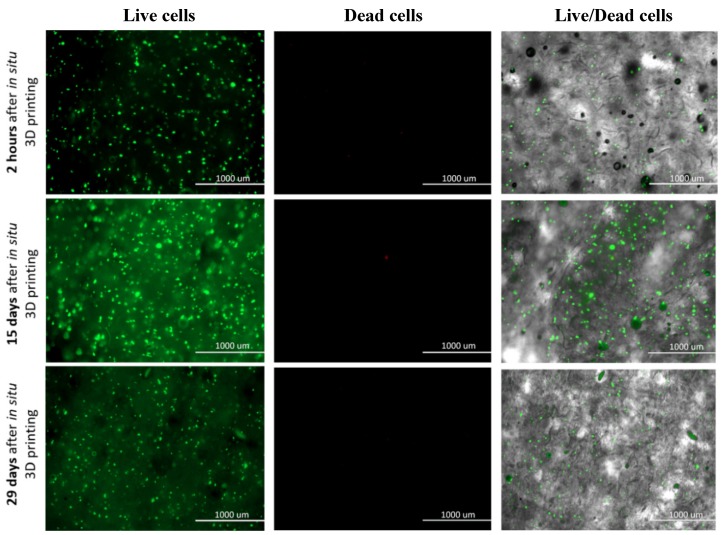
Live/Dead assay for 3D bioprinted hSF-Laden scaffolds (all time points are shown in the Supplementary Materials—Figures S2 and S3).

Based to the above mentioned, we evaluated the suitability of the prepared bioink with incorporated hSF cells and the effectiveness of the proposed bioprinting approach, for a rapid fabrication of a viable and physiologically relevant 3D dermis model. For this purpose, we performed the so-called Live/Dead assay, which presents a relatively simple, yet effective method, to test cell viability through respective staining of living (green) and dead (red) cells. The staining was carried out directly on the 3D hSF-laden scaffolds at predefined time intervals (2 h, 24 h, 48 h, 4 days, 6 days, 8 days, 12 days, 15 days, 25 days, and 29 days), and the obtained results are shown in Figure 5. The staining not only provides information about the cell’s general viability, but also related the latter with evaluation of potential harmful effects of the bioprinting process. For this purpose, we imaged the stained hSF-laden scaffolds also after 2 h. 

A homogeneous cell distribution was observed after encapsulating the hSF cells in the ALG/CMC/NFC hydrogel and the bioprinting of the prepared bioink in the form of 3D scaffolds. Such distribution implies an effective in situ incorporation and capability of this bioink to keep the cells in suspension. Even more importantly, the proposed hSF-laden bioink seems to be stable also in the cartridge during the bioprinting procedure. The hSF-laden bioinks properties (e.g., its enhanced stiffness) proved to protect the encapsulated hSF cells from any process-induced damages (e.g., shear stress, cross-linking procedure, etc.). Furthermore, cell viability of the hSF cells improved with time of their “culturing” in the 3D hSF-laden bioprinted scaffolds. This effect can be deduced from the decrease in the (already really small number) dead hSFs encapsulated in the bioink (seen through comparison of the micrographs between 15th and 29th day of culturing). Furthermore, the almost undiminished cell viability seen even after 29 days of culturing, indicates a complete lack of any potential toxic effect of the used base bioink components or their side products, which might occur during the scaffold degradation, for the mentioned period. The “apparent” lower fluorescence intensity after 29 days of culture (compared to the micrographs after 15 days), which might imply an overall lower cell viability, might be related to another positive observable effect during this period of culture. Namely, the somewhat increased homogenous distribution of cells, which is seen after 29 days, potentially indicates even an improved migration potential. This is another favorable effect, aiding regeneration, but will have to be further evaluated in the following studies. This last result is also in complete agreement with the results of degradation (some degradation due to ion exchange in ALG) and swelling (partial loosening of the main scaffolds internal structure), as discussed above. Micrographs that correspond to all time points, at which the Live/Dead assay was performed are shown in the Supplementary Materials (Figures S2 and S3).

The cellular activity (e.g., migration, proliferation, and differentiation) in a 3D microenvironment essentially depends on the integrated effects related to adhesion, as well as on the availability of sufficient space in the 3D structure, allowing cell migration and/or proliferation [58,59]. The obtained results from the Live/Dead staining suggest a favorable degradation rate of the hSF-laden scaffolds for further development of skin tissue models/substitutes. Namely, the observed formation of the cell aggregates with the prolonged culturing time, suggests that a continuous degradation of the ALG/CMC/NFC scaffolds, which is in turn beneficial for cell adhesion, propagation and aggregation, is present [52]. The formation of cell aggregates further indicates an increased cell proliferation and the establishment of intercellular communication, which plays a vital role in the inhibition of apoptosis (indirectly observed among others through the absence of red-dyed dead cells after 29 days of culturing) [60]. It is well known that mammalian cells are less prone to intensively proliferate in contact with ALG-based materials (despite the ALGs proven biocompatibility with various cells [61]) due to the hydrophilic nature of alginic acid, which adsorbs less protein, leading to reduced attachment and spreading, as well as due to an “unstable surface”, which results from potential ion exchange (Na^+^ and Ca^2+^; as described above). In this regard, several engineering approaches have been investigated to improve cell adhesion on alginate-based substrates. Recently, Salesa and colleagues [62], attempted nanotechnological strategy to overcome poor adhesion properties of alginate-based substrates, however without desired improvement of HACAT adhesion. Authors elucidate that this unfavorable outcome might be attributed to ionic cross-linking, which produced tight composite surfaces with one-dimensional and two-dimensional nanomaterials not exposed to cells. We hypothesize that in our study, the favorable cellular activity cannot be only assigned to 3D architecture of hSF-laden construct, but also to the excreted ECM proteins in ALG/CMC/NFC polymeric network. Therefore, the underlying hSFs with their excreted ECM proteins presumably modify the ALG/CMC/NFC scaffolds internal structure, and hence promote the attachment of “new” cells [60,63]. To summarize, the gathered results indicate that the hSF-laden bioink-based 3D bioprinted scaffolds not only promote cell aggregation and proliferation (as discussed above in relation to Figure 5, potentially even migration), but also support long-term cell growth, although further quantification of these results (e.g., multi-time point viability/metabolic activity testing), will be necessary in the future, to give further weight to this claim. As will be shown below (and in the Supplementary Materials, Figure S4) the same scaffolds have proven to serve also as effective substrates for keratinocytes attachment (formation of the epidermal skin layer). 

### 3.6. Future Work and Preliminary Results of the “Full Skin” Model Preparation

In this work, we developed a skin-cell loaded bioink formulation based on a polysaccharide (ALG/CMC/NFC) hydrogel matrix and hSF cells, which shows a positive trend in material–cell interactions. The subsequent positive effects on cellular activity can be attributed at least partially to the highly hydrated cellulose nanofibrils (NFC) and their morphological similarities with the skins native ECM components at the nanoscale level [18,28]. Following the printing steps used for fabrication of the 3D hSF-laden scaffolds, our objective was to construct a dermal 3D skin model, whereas the final goal is to prepare a functional bi-layered human skin model, composed of the herein prepared hSF-laden scaffold as the artificial dermis, covered with human skin-derived keratinocytes (e.g., immortalized HACAT cell line). Both cell lines are the primary sources of various ECM proteins and bioactive molecules, such as cytokines and growth factors that stimulate and coordinate tissue repair [10]. 

As we have already observed, it is important that the cell printing process of such a bi-layered skin model is done stepwise, as formation of the epidermal layer (composed of keratinocytes) should be done within a short interval after the fabrication of the dermal (underlying) structure. The herein used hydrogel matrix has proven to provide a highly suitable 3D environment for the encapsulation of the hSF cells, while it at the same time stabilized the prepared skin tissue model without any supportive materials. The upgrade of the prepared 3D hSF-laden scaffolds towards the full skin model, includes their submerging into cell culture media for 7–10 days to allow hSF to divide and multiply, resulting in production of appropriate ECM proteins [21,64] This step will be followed by seeding of keratinocytes onto the scaffolds. This co-culture construct must then be cultivated for another 5–10 days to enable attachment of keratinocytes onto hSF-laden scaffold, their proliferation and differentiation. The development of a confluent keratinocyte monolayer is crucial for the homogeneous epidermal stratification. Namely, patchy keratinocyte monolayers may result in the formation of immature keratinocyte layers that can easily peel off from the dermal substrate. Considering the latter, it is important to grow a rapid and uniform confluent monolayer before the onset of keratinocytes differentiation [65]. To achieve stratification, the so-formed 3D skin construct needs to be further matured by exposing it to an air-liquid interface for at least 10 days. In order to prove that a functionally and morphologically representative skin substitute has formed, histological analysis, to evaluate its phenotypical and morphological resemblance with that of the human skin, will be essential. During repair of cutaneous wounds, the differentiation of fibroblasts toward myofibroblasts with accompanying contraction is a significant contributor in scar formation [66]. Thus, to minimize the tissue deformation, the contractile forces of fibroblasts need to be more evenly dispersed. The latter can be gained with uniform gelling (cross-linking) conditions that prevent formation of heterogeneous regions of gelled and non-gelled hydrogel matrices [11]. By investigating the status of fibroblasts differentiation and the microarchitecture of the 3D printed ALG/CMC/NFC-bioink can shed some light about the potential of printed skin substitutes to reduce tissue contraction during wound healing. A proof of the suitability of the prepared model of the dermis through 3D printing of the developed bioink formulation, are the patches of the keratinocyte (epidermal layer) that formed on the surface of the base bioprinted dermal construct (see Supplementary Materials, Figure S4). However, to confirm bioactive features of our dermal construct to attract and retain a large number of keratinocytes, some additional cell adhesion assessment on hSF-laden should be performed by using, for example, an arbitrary index inversely proportional to the cell detachment kinetics [67]. Furthermore, scanning electron microscopy (SEM) was performed in order to indirectly evaluate the interaction between the keratinocytes attached to the scaffold surface. Figures S5–S7 in the Supplementary Materials (this describes also the details about the used SEM apparatus and the overall method used for this purpose) shows the SEM micrographs of the scaffold morphology (after different periods during growth of the cells in the scaffolds, Figure S5), which shows that the general morphology of the scaffolds is preserved for the whole experiment duration (29 days), as well as a close magnification of a keratinocyte cell, attached to the base scaffold surface (taken after 15 days of their growth on the scaffolds, Figure S6). These preliminary results are complementary to the other ones presented in this study, which altogether indicate the high promise of the developed formulation for preparation of representative human skin models.

## 4. Conclusions

Herein, we demonstrated the development of a human skin fibroblast (hSF)-laden, natural polysaccharide (e.g., ALG, CMC, and NFC)-based bioink and its 3D bioprinting, with the intention to develop novel drug testing platforms, as well as concrete treatment solutions in wound healing. The biologically relevant 3D environment of the proposed bioinks composition, which exhibits a similar morphology to collagen (and important product of the skins native extracellular matrix (ECM)), provides the hSF with surroundings that resembles their natural environment. The excellent printability of the developed hSF-laden bioink, make it possible to 3D bioprint complex scaffolds with a controlled cell density and defined porosity. The 3D hSF-laden scaffolds displayed shape and size stability after the bioprinting process and after culturing for up to 29 days. Moreover, characterization of the 3D hSF-laden scaffolds proved a high incorporated cell viability, cell proliferation, and a tendency to form agglomerates during culturing in a 3D environment (in the scaffolds). Further optimization of this biofabrication platform can potentially facilitate the construction of a more complex bi-layered in vitro skin model. Such models can significantly contribute to our better understanding of the epidermal biology, which might not only aid the advancement of the fabrication of superior skin grafts but can also serve as powerful tools for testing topical and transdermal formulations with incorporated drugs, reducing the reliance on animal models. Overall, bioactive features coupled with proper printability, make the developed hSF-laden bioink a promising biomaterial for skin tissue engineering, a drug testing platform, and many other biomedical applications.

## Supplementary Materials

The following are available online at https://www.mdpi.com/2079-4991/10/4/733/s1, Figure S1: scaffold model and photographs of printed ink and bioink formulations; Figures S2 and S3: Live/Dead assay of the 3D bioprinted hSF-laden scaffolds at given time periods (2 h, 24 h, 48 h, 4 days, 6 days, 8 days, 12 days, 15 days, 25 days and 29 days); Figure S4: Viability of the simple bilayer *in vitro* skin model: (a) photographs of 3D bioprinted hSF-laden scaffolds seeded with HACAT cell line; (b) micrographs of grown HACAT cell on 3D bioprinted hSF-laden scaffolds; (c) Live/Dead assay of the simple bilayer in vitro skin model; Figures S5 and S6: SEM micrographs of the scaffold morphology: (a) top views of the scaffolds; (b) side views of the scaffolds. Figure S7: SEM micrograph of a keratinocyte cell attached to the scaffold surface. Details about the used SEM method is described in Supplementary Materials.
