# Supplementary material for: Polysaccharide-Based Bioink Formulation for 3D Bioprinting of an In Vitro Model of the Human Dermis
Source: Nanomaterials (Basel). 2020 Apr 11;10(4):733. doi: 10.3390/nano10040733 (PMC7221685; doi:10.3390/nano10040733)
Supplement: Supplementary file 1 [file nanomaterials-10-00733-s001.pdf]

nanomaterials

Supplementary Materials: Polysaccharide-Based Bioink Formulation for 3D Bioprinting of an In Vitro Model of the Human Dermis

Tanja Zidarič [1,*], Marko Milojević [1], Lidija Gradišnik [1], Karin Stana Kleinschek [2,3,4], Uroš Maver [1,5,*] and Tina Maver [1,2,*]

[1] Institute of Biomedical Sciences, Faculty of Medicine, University of Maribor, Taborska ulica 8, 2000 Maribor, Slovenia; marko.milojevic1@um.si (M.M.); lidija.gradisnik@um.si (L.G.)
[2] Laboratory for Characterization and Processing of Polymers (LCPP), Faculty of Mechanical Engineering, University of Maribor, Smetanova ulica 17, 2000 Maribor, Slovenia; karin.stanakleinschek@tugraz.at
[3] Chemical and Process Engineering and Biotechnology, Institute of Chemistry and Technology of Biobased Systems, Faculty of Technical Chemistry, Graz University of Technology, Stremayrgasse 9, 8010 Graz, Austria
[4] Institute of Automation, Faculty of Electrical Engineering and Computer Science, University of Maribor, Koroška cesta 46, 2000 Maribor, Slovenia
[5] Department of Pharmacology, Faculty of Medicine, University of Maribor, Taborska ulica 8, 2000 Maribor, Slovenia
* Correspondence: tanja.zidaric@um.si (T.Z.); uros.maver@um.si (U.M.); tina.maver@um.si (T.M.)

1. Model of the scaffold and photographs of printed ink and bioink formulations.

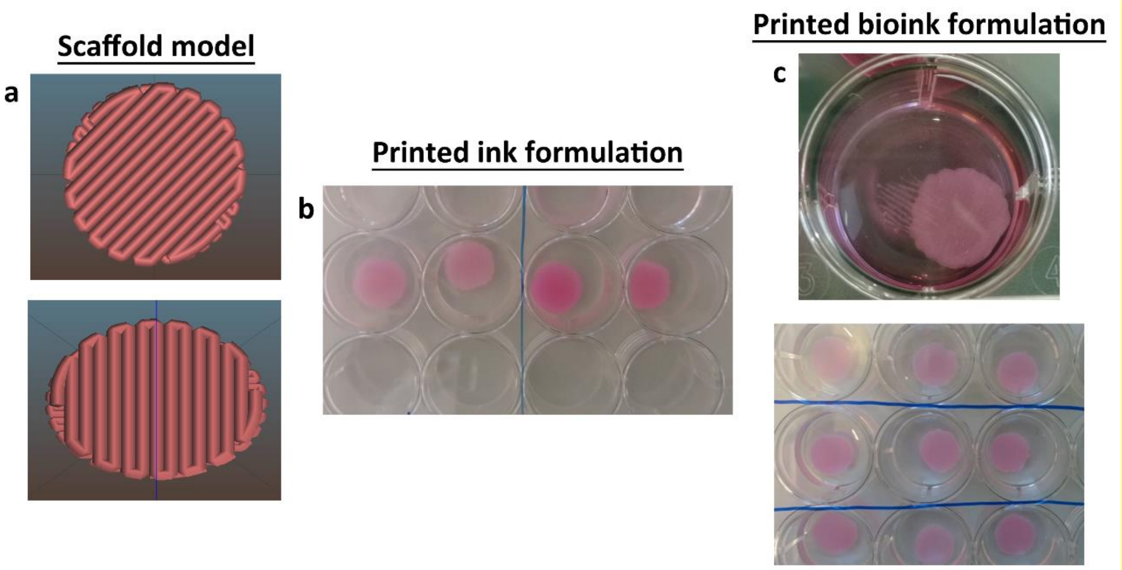

Figure S1. a) Model of the 3D printed scaffold, **b**) printed bioink formulation in a P12 well plate, and **c**) printed bioink formulation.

2. Live/Dead assay of the 3D bioprinted hSF-laden scaffolds.

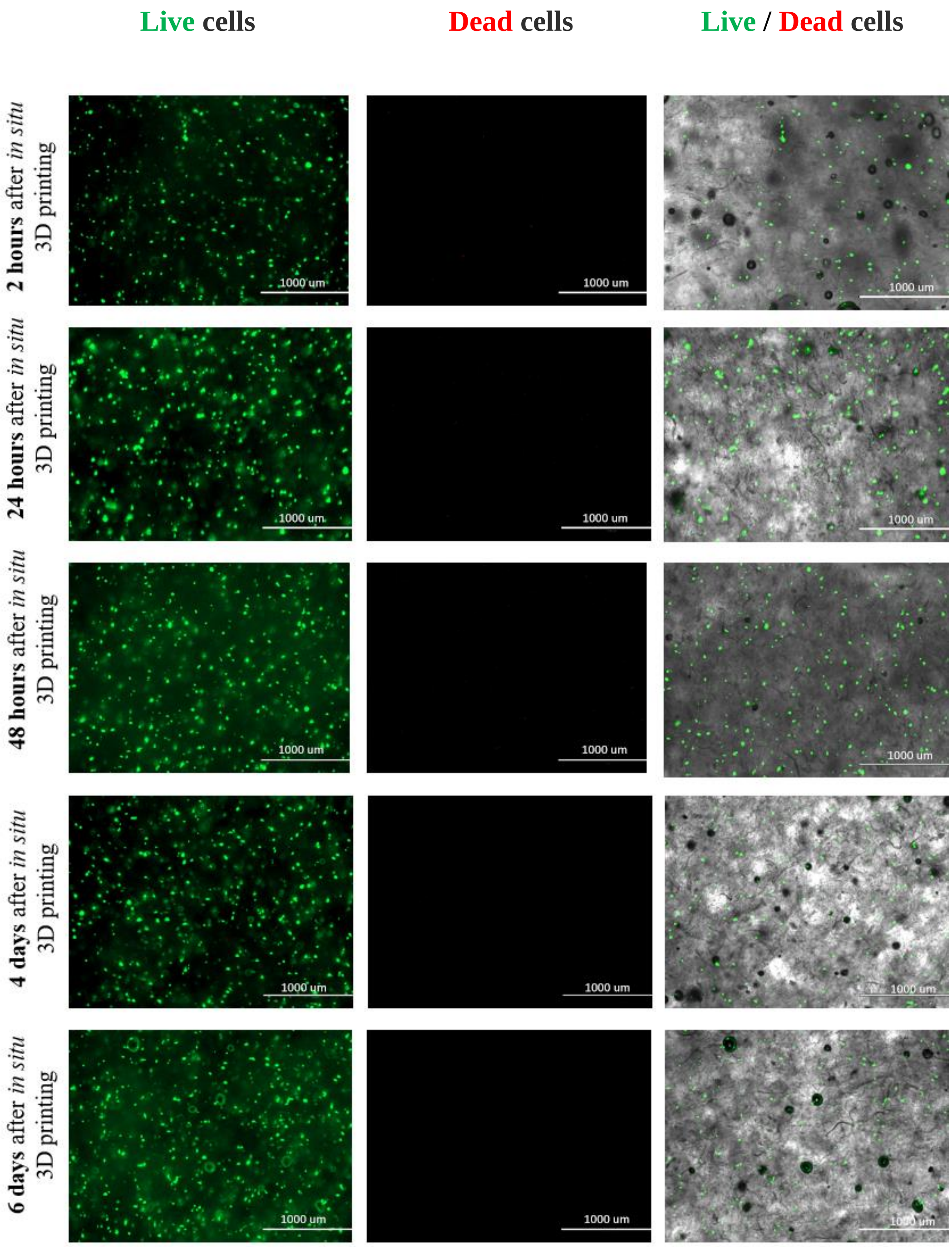

Figure S2. Live/Dead assay of the 3D bioprinted hSF-laden scaffolds at given time points.

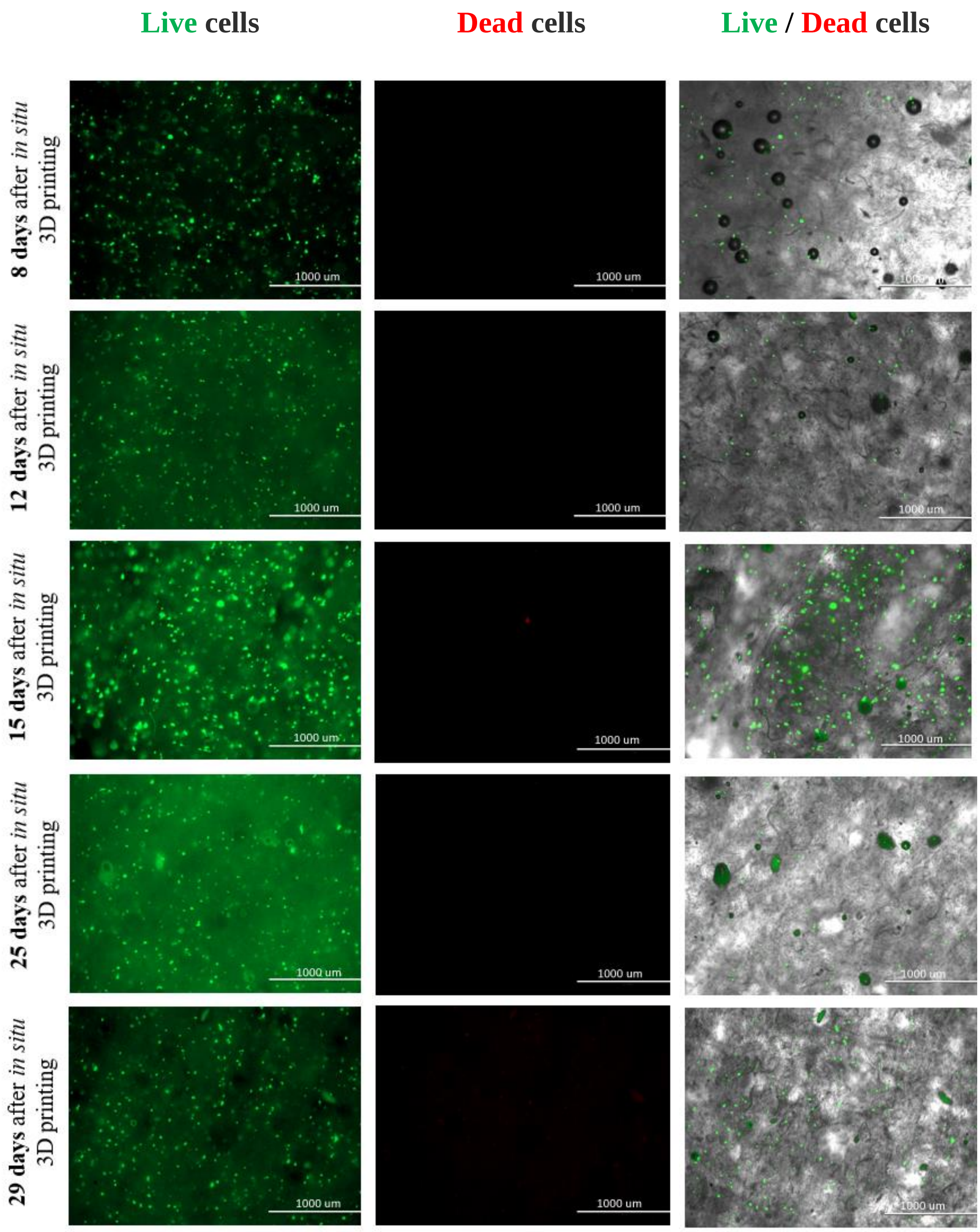

Figure S3. Live/Dead assay of the 3D bioprinted hSF-laden scaffolds at given time points (continued).

3. Viability of the simple bilayer *in vitro* skin model.

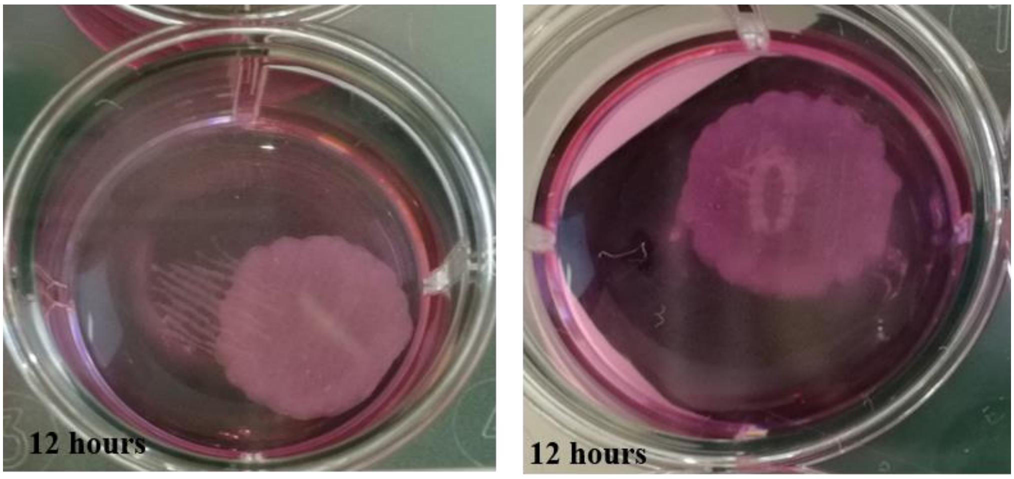

(**a**)

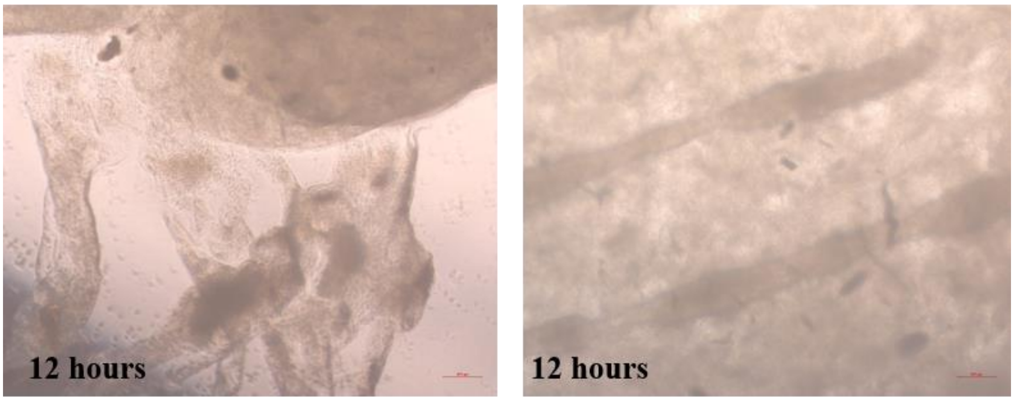

(**b**)

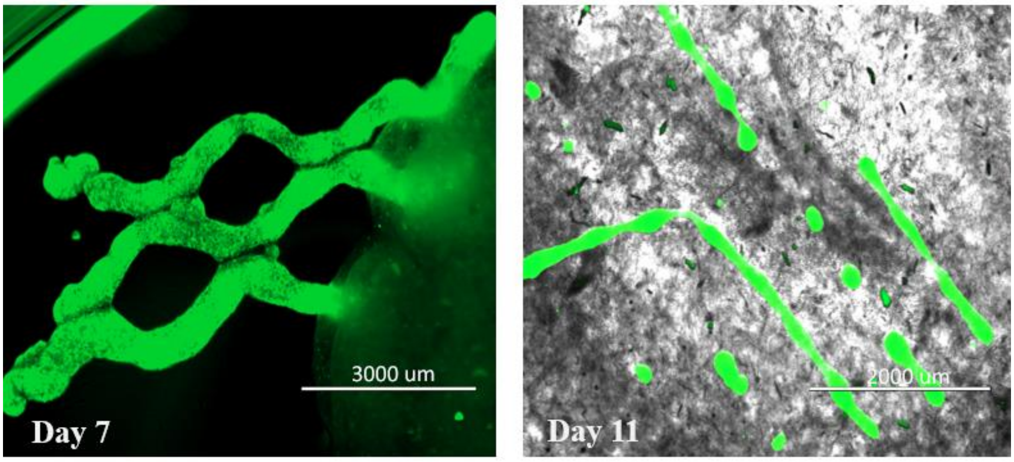

(**c**)

Figure S4. Viability of the simple *in vitro* skin model: (**a**) the photographs of 3D bioprinted hSF-laden scaffolds seeded with HACAT cell line (formed monolayer can be clearly seen); (**b**) the micrographs of grown HACAT cells on 3D bioprinted hSF-laden scaffolds; (**c**) Live/Dead assay performed on the simple bilayer *in vitro* skin model (epidermis/HACAT monolayer, dermis/hSF-laden scaffold).

4. Scanning electron microscopy (SEM) method description.

The sample morphology was analysed using Field Emission Scanning Electron Microscopy (FESEM, Carl Zeiss FE-SEM SUPRA 35 VP electron microscope, Zeiss, Germany). Prior to measurements, all samples were lyophilized and individually placed on aluminium SEM sample holders using double-side conductive carbon tape. The samples were sputtered using a Benchtop Turbo sputtering device (Dentum Vacuum, Moorestown, NJ, USA) with a thin layer of palladium. The SEM images were recorded with an accelerating voltage of 1keV at an approximately 4.5 mm working distance.

4 days

6 days

8 days

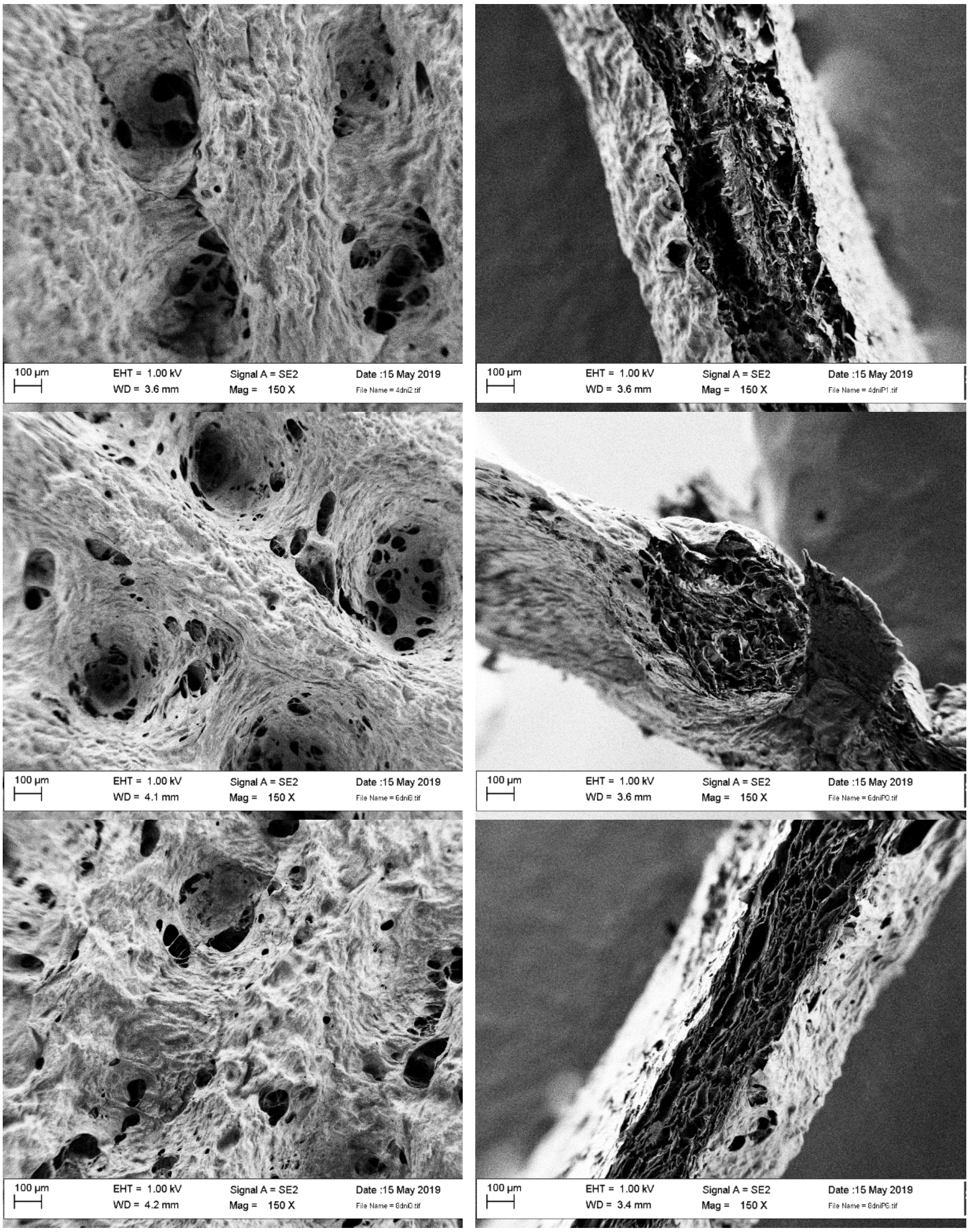

(**a**) (**b**)

Figure S5. SEM micrographs showing the scaffold morphology (after 4, 6, and 8 days of cell growth): (**a**) the micrographs show the top views of the scaffolds; (**b**) the micrographs show the side views of the scaffolds. As can be seen, the general morphology of the scaffolds is preserved in both direction for the whole experiment duration.

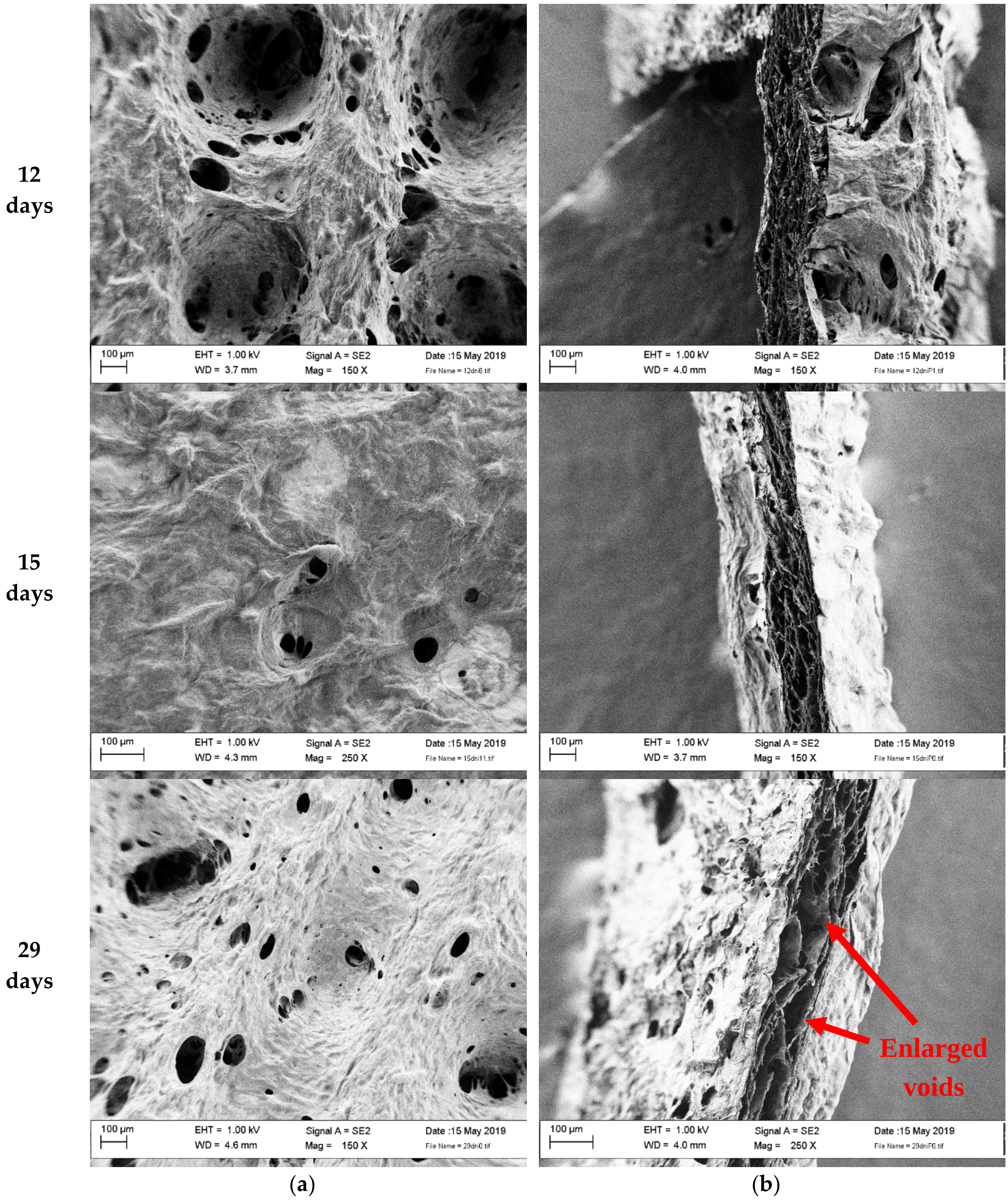

Figure S6. SEM micrographs showing the scaffold morphology (after 12, 15, and 29 days of cell growth): (**a**) the micrographs show the top views of the scaffolds; (**b**) the micrographs show the side views of the scaffolds (continued). The red arrows show an apparent enlargement of the internal spacings (voids) inside the scaffolds, which might contribute to promotion of cell migration and proliferation.

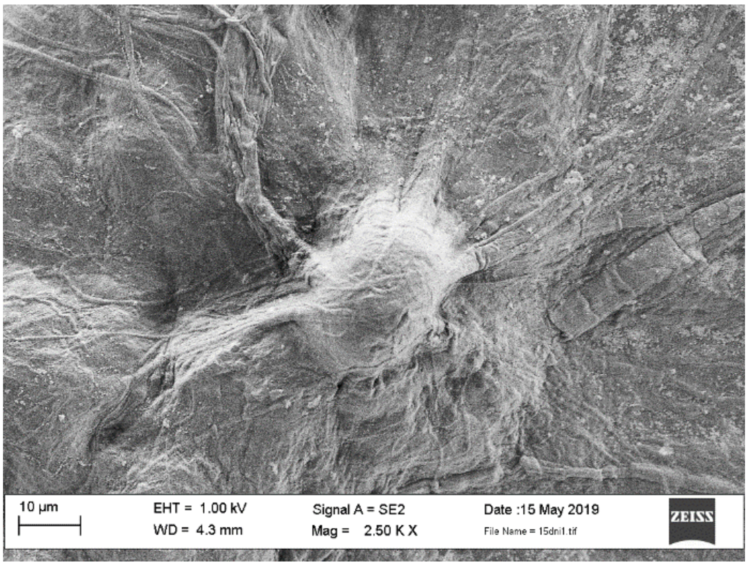

Figure S7. SEM micrograph showing a high magnification of a keratinocyte cell, attached to the base scaffold surface (taken on a scaffold after 15 days of cell growth).
